# Predictive factors of progression to severe COVID-19

**DOI:** 10.1515/med-2020-0184

**Published:** 2020-08-28

**Authors:** Yi-Hong Zhou, Huan Li, Yuan-Yuan Qin, Xiao-Feng Yan, Yan-Qiu Lu, Hong-Lan Liu, Si-Kuan Ye, Yan Wan, Lu Zhang, Vijay Harypursat, Yaokai Chen

**Affiliations:** Department of Infectious Diseases, Chongqing Public Health Medical Center, No. 109 Baoyu Road, Shapingba, Chongqing, 400036, China; Department of Tuberculosis, Chongqing Public Health Medical Center, Chongqing, China

**Keywords:** Coronavirus, SARS-CoV-2, COVID-19, predictive, progression

## Abstract

**Aim:**

Early diagnosis and treatment are crucial for the survival of severe Coronavirus Disease 2019 (COVID-19) patients, but data with regard to risk factors for disease progression from milder COVID-19 to severe COVID-19 remain scarce.

**Methods:**

We conducted a retrospective analysis on 116 patients.

**Results:**

Three factors were observed to be independently associated with progression to severe COVID-19 during 14 days after admission: (a) age 65 years or older (hazard ratio [HR] = 8.456; 95% CI: 2.706–26.426); (b) creatine kinase (CK) ≥ 180 U/L (HR = 3.667; 95% CI: 1.253–10.733); and (c) CD4+ T-cell counts <300 cells/µL (HR = 4.695; 95% CI: 1.483–14.856). The difference in rates of severe COVID-19 development was found to be statistically significant between patients aged 65 years or older (46.2%) and those younger than 65 years (90.2%), between patients with CK ≥ 180 U/L (55.6%) and those with CK < 180 U/L (91.5%), and between patients with CD4+ T-cell counts <300 cells/µL (53.8%) and those with CD4+ cell counts ≥300 cells/µL (83.2%).

**Conclusions:**

Age ≥ 65 years, CK ≥ 180 U/L, and CD4+ T-cell counts <300 cells/µL at admission were risk factors independently associated with disease progression to severe COVID-19 during 14 days after admission and are therefore potential markers for disease progression in patients with milder COVID-19.

## 1 Introduction

Severe acute respiratory syndrome coronavirus 2 (SARS-CoV-2) is a novel coronavirus that emerged in Wuhan, the provincial capital of Hubei Province, China, in December 2019 [1]. The virus is known to spread with ease from person to person among close contacts [2] and may cause an acute respiratory illness, which has been named Coronavirus Disease 2019 (COVID-19). Most patients with SARS-CoV-2 infection develop mild to moderate upper respiratory tract symptoms, whereas others may develop severe respiratory distress, systemic sepsis, septic shock, and death [3,4,5]. Some patients present with fever and respiratory symptoms such as cough and shortness of breath, and others may present with gastrointestinal symptoms including diarrhea, vomiting, and abdominal pain [6]. In addition, some atypical symptoms such as altered mental status, symptoms of stroke, and olfactory and gustatory dysfunctions have been described [7,8]. Although patients with mild to moderate COVID-19 usually have a good prognosis, severe COVID-19 is associated with high mortality.

Early diagnosis and treatment are crucial for the survival of severe COVID-19 patients. As most patients who develop severe COVID-19 start with mild symptoms and later progress to severe disease, it is imperative to identify potential risk factors for disease progression in this population. This may help healthcare providers timeously identify patients with disease progression potential, thus facilitating early diagnosis and treatment of severe COVID-19. However, data with regard to potential risk factors for disease progression from mild or moderate COVID-19 to severe COVID-19 outside Wuhan remain scarce in the published literature and therefore warrant further investigation.

Our infectious disease hospital began admitting COVID-19 patients from January 24, 2020, and over 200 COVID-19 patients had been admitted up until February 20, 2020. The majority were diagnosed with mild or moderate COVID-19 at admission. However, a subgroup of these milder COVID-19 patients progressed to severe COVID-19 during their hospital stay, whereas others stabilized and recovered. In the present study, we retrospectively analyzed the clinical data of patients admitted to our hospital with milder COVID-19, including those who progressed to severe disease after admission. Our objective was to investigate the presence of potential risk factors associated with disease severity progression in the natural history of COVID-19.

## 2 Material and methods

### 2.1 Ethics, consent, and permissions

This study was approved by the Ethics Committee of Chongqing Public Health Medical Center (2020-003-01-KY). Informed consent was waived as all data were retrospective and were collected anonymously.

### 2.2 Patient enrollment and data collection

We included all patients aged 18 or older who had a confirmed diagnosis of mild or moderate COVID-19, who were admitted to Chongqing Public Health Medical Center, China, from January 24, 2020, to February 7, 2020. We transcribed demographics, epidemiological information, clinical manifestations, and clinical outcomes of eligible patients from the electronic hospital medical record system onto case record forms. Laboratory test results including blood gas analysis, hematological analysis, C-reactive protein, coagulation tests, myocardial enzymes, clinical chemistry, and lymphocyte subsets were also extracted from the records and recorded.

Patients exhibiting one or more of the following conditions were classified as having severe COVID-19: (a) respiratory distress (≥30 breaths/min); (b) oxygen saturation ≤93% at rest; (c) arterial partial pressure of oxygen (PaO_2_)/fraction of inspiration O_2_ (FiO_2_) ≤300 mmHg (1 mmHg = 0.133 kPa); (d) respiratory failure requiring mechanical ventilation; (e) development of septic shock; and (f) critical organ failure requiring ICU care. Patients not meeting the aforementioned criteria were classified as mild or moderate COVID-19 cases and referred to as “milder” cases as a stratification category to clearly differentiate between milder and severe cases of COVID-19 in our data analysis.

### 2.3 Statistical analysis

All analyses were performed using Statistical Package for the Social Sciences software, Version 19.0 (IBM SPSS Statistics, Chicago, IL, USA). Categorical variables were described as frequency rates and percentages and compared via the Chi-squared test or the Fisher exact test as appropriate. Continuous variables were described using mean, median, and interquartile range (IQR) values. Mean values for continuous variables were compared using independent group *t*-tests when the data were normally distributed; otherwise, the Mann–Whitney test was used. Statistical significance was assumed when *p*-values less than 0.05 were calculated. Furthermore, time to developing severe COVID-19 was analyzed over the duration of 14 days of hospitalization by the Kaplan–Meier method. The hypothesis test was two tailed, with a *p* ≤ 0.05 indicative of statistical significance. Cox regression was applied to estimate the unadjusted hazard ratios (HRs) of risk factors for disease progression during 14 days of hospitalization, and adjusted HRs were identified by using a forward stepwise approach.

## 3 Results

### 3.1 Patient characteristics

A total of 130 patients with mild or moderate COVID-19 were admitted to our hospital from January 24, 2020, to February 7, 2020. After excluding four patients younger than 18 years and ten patients diagnosed with severe COVID-19 at admission, a total of 116 patients were included for the analysis in this study. Of them, 17 patients (14.7%) eventually developed severe disease, while 99 patients (85.3%) did not meet the criteria for diagnosis of severe COVID-19 during 14 days of hospitalization.

As depicted in Table 1, patients who developed severe COVID-19 were significantly older (59 years [IQR, 50–70] vs 41 years [IQR, 35–54], *p*  < 0.001) compared with those who did not develop severe COVID-19. In the 17 patients who went on to develop severe COVID-19 during 14 days of hospitalization, the median duration from the symptom onset to the diagnosis of severe COVID-19 was 12 days (IQR, 10–15), and the median duration from hospital admission to diagnosis of severe COVID-19 was 6 days (IQR, 4–9).

**Table 1:** Patient characteristics at hospital admission

Characteristics	Total (*n* = 116)	No progression to severe COVID-19 (*n* = 99)	Progression to severe COVID-19 (*n* = 17)	*p*
Medical age, median (IQR), years	46 (36–56)	41 (35–54)	59 (50–70)	<0.001
Gender, *n* (%)				
Male	59	51 (51.5)	8 (47.1)	0.734
Female	57	48 (48.5)	9 (52.9)	
Married, *n* (%)				
Yes	98	82 (82.8)	16 (94.1)	0.409
No	18	17 (17.2)	1 (5.9)	
Smoking, *n* (%)				
Yes	21	17 (17.2)	4 (23.5)	0.773
No	95	82 (82.8)	13 (76.5)	
BMI	23.69 ± 2.92	23.6 ± 2.92	24.11 ± 2.94	0.527
Symptoms at admission, *n* (%)				
Yes	106	90 (90.9)	16 (94.1)	1.000
No	10	9 (9.1)	1 (5.9)	
History of stay in Wuhan, *n* (%)				
Yes	37	34 (34.3)	3 (17.6)	0.172
No	79	65 (65.7)	14 (82.4)	
Diabetes, *n* (%)				
Yes	9	7 (7.1)	2 (13.3)	0.859
No	107	92 (92.9)	15 (86.7)	
Hypertension, *n* (%)				
Yes	14	11 (11.1)	3 (17.6)	0.718
No	102	88 (88.9)	14 (82.4)	
Time from symptom onset to hospital admission, median (IQR), d	4 (2–7)	4 (2–7)	5 (4–7)	0.224
Time from symptom onset to severe COVID-19, median (IQR), d	—	—	12 (10–15)	—
Time from hospital admission to severe COVID-19, median (IQR), d	—	—	6 (4–9)	—

BMI: body mass index; IQR: interquartile range.

### 3.2 Comparison of clinical manifestations between the two groups

In our cohort of 116 patients, fever was the most common symptom at illness onset, occurring in 67.2% of patients in our cohort, followed by cough (59.5%), sputum production (37.9%), fatigue (27.6%), and anorexia (23.3%). In addition, some patients presented with atypical symptoms including heart palpitations (0.9%), xerostomia (0.9%), hemoptysis (0.9%), hyposmia (0.9%), and low back pain (1.7%). There were no significant differences in clinical symptoms between patients who developed severe COVID-19 and those who did not during 14 days of hospitalization after admission in our cohort (Table 2).

**Table 2:** Clinical manifestations of patients with COVID-19 at hospital admission

Signs and symptoms	Total	No progression to severe COVID-19	Progression to severe COVID-19	*p*
Fever, *n* (%)				
Yes	78	67 (67.7)	11 (64.7)	0.809
No	38	32 (32.2)	6 (35.3)	
Rigors, *n* (%)				
Yes	7	7 (7.1)	0 (0.0)	0.562
No	109	92 (92.9)	17 (100)	
Fatigue, *n* (%)				
Yes	32	28 (28.3)	4 (23.5)	0.911
No	84	71 (71.7)	13 (76.5)	
Cough, *n* (%)				
Yes	69	58 (58.6)	11 (64.7)	0.635
No	47	41 (41.4)	6 (35.3)	
Dyspnea, *n* (%)				
Yes	3	1 (1)	2 (11.7)	0.056
No	113	98 (99.0)	15 (88.2)	
Sputum, *n* (%)				
Yes	44	36 (36.4)	8 (47.1)	0.401
No	72	63 (63.6)	9 (52.9)	
Sore throat, *n* (%)				
Yes	20	17 (17.2)	3 (17.6)	1.000
No	96	82 (82.8)	14 (82.4)	
Xerostomia, *n* (%)				
Yes	1	1 (1.0)	0 (0.0)	1.000
No	115	98 (99.0)	17 (100.0)	
Hemoptysis, *n* (%)				
Yes	1	0 (0.0)	1 (5.9)	0.147
No	115	99 (100)	16 (94.1)	
Palpitations, *n* (%)				
Yes	1	0 (0.0)	1 (5.9)	0.147
No	115	99 (100.0)	16 (94.1)	
Myalgia, *n* (%)				
Yes	10	9 (9.2)	1 (6.7)	1.000
No	106	89 (90.8)	14 (93.3)	
Arthralgia, *n* (%)				
Yes	4	3 (3.0)	1 (5.9)	0.474
No	112	96 (97.0)	16 (94.1)	
Low back pain, *n* (%)				
Yes	2	2 (2.0)	0 (0.0)	1.000
No	114	97 (98.0)	17 (100.0)	
Abdominal pain, *n* (%)				
Yes	4	3 (3.0)	1 (5.9)	0.474
No	112	96 (97.0)	16 (94.1)	
Nausea and vomiting, *n* (%)				
Yes	4	3 (3.0)	1 (5.9)	0.474
No	112	96 (97.0)	16 (94.1)	
Diarrhea, *n* (%)				
Yes	10	9 (9.1)	1 (5.9)	1.000
No	106	90 (90.9)	16 (94.1)	
Anorexia, *n* (%)				
Yes	27	22 (22.2)	5 (29.4)	0.736
No	89	77 (77.8)	12 (70.6)	
Headache, *n* (%)				
Yes	15	12 (12.1)	3 (17.6)	0.813
No	101	87 (87.9)	14 (82.4)	
Dizziness, *n* (%)				
Yes	14	11 (11.1)	3 (17.6)	0.718
No	102	88 (88.9)	14 (82.4)	
Hyposmia, *n* (%)				
Yes	1	1 (1.0)	0 (0.0)	1.000
No	115	98 (99.0)	17 (100.0)	
Asymptomatic, *n* (%)				
Yes	10	9 (9.1)	1 (5.9)	1.000
No	106	90 (90.9)	16 (94.1)	
Moist rales, *n* (%)				
Yes	9	6 (6.1)	3(33.3)	0.246
No	107	93 (93.9)	14 (82.4)	
Median pulse, mean ± SD, beats/min	90.06 ± 13.065	90.40 ± 13.293	88.06 ± 11.808	0.465
Median systolic blood pressure, median (IQR), mm Hg	127.25 ± 15.148	126.69 ± 15.116	130.53 ± 15.371	0.336

SD: standard deviation.

### 3.3 Comparison of laboratory test results between the two groups

Compared with patients who had milder COVID-19, those who developed severe COVID-19 after admission had significantly lower lymphocyte counts, platelet counts, estimated glomerular filtration rates (eGFRs), CD4+ T-cell counts, CD8+ T-cell counts, and PaO_2_/FiO_2_ ratios, and significantly higher C-reactive protein levels, lactate dehydrogenase levels, aspartate transaminase levels, and beta 2-microglobulin levels (Table 3).

**Table 3:** Laboratory findings of patients with COVID-19 at hospital admission

Laboratory values	Number of patients	Total	No progression to severe COVID-19	Progression to severe COVID-19	*p*
White blood cell count (×10^9^/L)	115	4.84 (3.87–5.84)	4.81 (3.85–6.05)	4.96 (3.89–5.54)	0.741
Neutrophil count (×10^9^/L)	114	2.87 (2.00–3.95)	2.77 (1.98–3.95)	3.41 (2.55–4.12)	0.310
Lymphocyte count (×10^9^/L)	115	1.32 (1.060–1.76)	1.45 (1.18–1.78)	1.03 (0.74–1.28)	0.002
Platelet count (×10^9^/L)	115	163 (128–212)	169.5 (132.25–214.00)	136 (96–174)	0.029
Hemoglobin (g/L)	115	136 (125–147)	137.5 (124.75–148)	128 (122–139)	0.117
C-reactive protein (mg/L)	112	9.36 (2.96–26.26)	7.99 (2.87–21.57)	25.55 (10.03–70.17)	0.002
D-dimer (mg/L)	112	0.16 (0.10–0.26)	0.14 (0.09–0.25)	0.21 (0.12–0.3)	0.157
CK (U/L)	112	74.5 (49.5–128)	70.5 (47.5–108.25)	149 (53.25–299)	0.088
LDH (U/L)	116	195 (164–251)	190 (163–237)	273 (185.5–310.5)	0.003
ALT (U/L)	116	21 (13.25–31.75)	21 (13–31)	21 (15–52)	0.290
AST (U/L)	116	24 (19–31)	22 (18–28)	33 (25–46.5)	0.002
Albumin (g/L)	116	42.236 ± 3.657	42.273 ± 3.544	40.613 ± 3.649	0.095
Total bilirubin (µmol/L)	116	13.05 (9.625–19.150)	12.7 (9.6–17.8)	17.6 (11.2–22.6)	0.102
Creatinine (µmol/L)	115	70.1 (59.3–84)	69.65 (58.08–83.55)	70.1 (61–89.35)	0.555
Beta 2-microglobulin (mg/L)	114	2.57 (2.15–3.05)	2.51 (2.14–2.95)	3.34 (2.53–3.79)	0.004
eGFR	115	98.36 ± 18.83	100.51 ± 18.177	83.94 ± 18.164	0.006
CD4+ T-cell counts (cells/µL)	85	424 (264–594)	478 (322–606)	243.5 (223–290.75)	<0.001
CD8+ T-cell counts (cells/µL)	83	316 (207–459)	359 (231–490)	159.5 (126–313.25)	0.003
CD4+ T-cell counts/CD8+ cell counts	83	1.31 (0.98–1.75)	1.36 (1.01–1.73)	1.13 (0.76–1.97)	0.403
PaO_2_/FiO_2_ (mmHg)	74	414.28 (365.48–455.54)	419 (376.19–471.42)	366.67 (321.67–413.14)	0.005

LDH: lactate dehydrogenase; ALT: alanine aminotransferase; AST: aspartate aminotransferase; eGFR: estimated glomerular filtration rate; PaO_2_: partial pressure of oxygen; PaO_2_/FiO_2_: partial arterial oxygen concentration/inspired oxygen faction.

### 3.4 Comparison of therapeutic interventions between the two groups

The proportion of antibiotic use in patients who developed severe COVID-19 was significantly higher than in patients who did not develop severe COVID-19 (35.3% vs 10.1%, *p* = 0.016) during 14 days of hospitalization. However, we found no statistical correlation in the relative use of lopinavir/ritonavir, ribavirin, and traditional Chinese medicine between the two groups of patients, as presented in Table 4.

**Table 4:** Treatment of COVID-19 patients within 14 days after admission

Characteristics	Total	No progression to severe COVID-19	Progression to severe COVID-19	*P*
LPV/r, *n* (%)				
Yes	88	74 (74.7)	14 (82.4)	0.711
No	28	25 (25.3)	3 (17.6)	
Ribavirin, *n* (%)				
Yes	46	42 (42.4)	4 (23.5)	0.141
No	70	57 (57.6)	13 (76.5)	
Antibiotics, *n* (%)				
Yes	16	10 (10.1)	6 (35.3)	0.016
No	100	89 (89.9)	11 (64.7)	
TCM, *n* (%)				
Yes	30	26 (26.3)	4 (23.5)	1.000
No	86	73 (73.7)	13 (76.5)	

LPV/r: lopinavir/ritonavir; TCM: traditional Chinese medicine.

### 3.5 Independent risk factors for progression to severe COVID-19

All variables with a *p* ≤ 0.1 in the univariate analysis, other than lymphocyte counts, were included in a Cox proportional hazards model and adjusted for symptoms at admission, ribavirin use, lopinavir/ritonavir use, comorbid diabetes, and comorbid hypertension. We did not include lymphocyte counts in this model to avoid the possible multicollinearity effect on CD4+ T-cell counts.

Three factors were found to be independently associated with progression to severe COVID-19 (Table 5) during 14 days of hospitalization after admission, and these factors are as follows: (a) age 65 years or older (HR = 8.456; 95% CI: 2.706–26.426; *p* < 0.001); (b) creatine kinase (CK) ≥ 180 U/L (HR = 3.667; 95% CI: 1.253–10.733; *p* = 0.018); and (c) CD4+ T-cell counts <300 cells/µL (HR = 4.695; 95% CI: 1.483–14.856; *p* = 0.009).

**Table 5:** Independent risk factors for progression to severe COVID-19

	Total	Progression to severe COVID-19	*p*	HR	95% CI	*p*	Adjusted HR	95% CI
Age								
<65 years	103	10		1			1	
≥65 years	13	7	<0.001	8.226	3.102–21.817	<0.001	8.456	2.706–26.426
Symptoms at admission, *n*								
No	10	1		1				
Yes	106	16	0.637	1.628	0.216–12.274			
BMI								
<24	55	6		1				
≥24	42	10	0.093	2.382	0.865–6.557			
Unknown	19	1	0.480	0.466	0.056–3.873			
Ribavirin, *n*								
Yes	46	4		1				
No	70	13	0.141	2.323	0.757–7.125			
LPV/r, *n*								
Yes	88	14		1				
No	28	3	0.476	1.574	0.452–5.477			
Antibiotics, *n*								
No	100	11		1				
Yes	16	6	0.006	3.996	1.476–10.821			
Dyspnea, *n*								
No	113	15		1				
Yes	3	2	0.004	9.015	2.027–40.092			
Diabetes, *n*								
Yes	9	2		1				
No	107	15	0.477	0.585	0.134–2.561			
Hypertension, *n*								
Yes	14	3		1				
No	102	14	0.409	0.591	0.170–2.058			
Platelet count								
≥100 × 10^9^/L	104	13		1				
<100 × 10^9^/L	11	4	0.038	3.273	1.066–10.051			
Unknown	1	0	0.988	0	0			
C-reactive protein								
<20 mg/L	74	6		1				
≥20 mg/L	38	11	0.005	4.166	1.540–11.274			
Unknown	4	0	0.985	0	0			
CK								
<180 U/L	94	8		1			1	
≥180 U/L	18	8	<0.001	6.575	2.458–17.590	0.018	3.667	1.253–10.733
Unknown	4	1	0.311	2.927	0.366–23.409	0.666	1.693	0.156–18.427
LDH								
<250 U/L	87	7		1				
≥250 U/L	29	10	0.001	5.06	1.925–13.305			
AST								
<40 U/L	100	12		1				
≥40 U/L	16	5	0.052	2.810	0.989–7.981			
Albumin								
≥40 g/L	87	9		1				
<40 g/L	29	8	0.026	2.954	1.139–7.660			
Beta-2 microglobulin								
<28 mg/L	68	5		1				
≥28 mg/L	46	12	0.009	3.987	1.404–11.326			
Unknown	2	0	0.983	0.000	0.000			
eGFR								
≥100	59	4		0.264				
<100	56	13	0.020	3.793	1.236–11.638			
Unknown	1	0	0.988	0	0			
PaO_2_/FiO_2_								
≥400 mmHg	41	3		1				
<400 mmHg	33	10	0.015	4.941	1.359–17.968			
Unknown	42	4	0.747	1.280	0.286–5.719			
CD4+ T-cell counts								
≥300/µL	59	4		1			1	
<300/µL	26	12	<0.001	8.778	2.825–27.275	0.009	4.695	1.483–14.856
Unknown	31	1	0.005	0.467	0.052–4.177	0.461	0.397	0.034–4.626
CD8+ T-cell counts								
≥238/µL	56	7						
<238/µL	27	9	0.028	3.022	1.125–8.118			
Unknown	33	1	0.170	0.230	0.028–1.872			

BMI: body mass index; LPV/r: lopinavir/ritonavir; CK: creatine kinase; LDH: lactate dehydrogenase; AST: aspartate aminotransferase; PaO_2_/FiO_2_: partial arterial oxygen concentration/inspired oxygen faction; eGFR: estimated glomerular filtration rate.

### 3.6 Relationship between the number of risk factors considered (age > 65, CK ≥ 180, CD4+ T-cell counts <300) and progression to severe COVID-19

The number of risk factors considered (age > 65, CK ≥ 180, and CD4+ cell counts <300) was included in a Cox proportional hazards model and adjusted for symptoms at admission, including body mass index, ribavirin use, lopinavir/ritonavir use, antibiotic use, dyspnea, comorbid diabetes, comorbid hypertension, platelet count, C-reactive protein, lactate dehydrogenase, aspartate aminotransferase, albumin, Beta-2 microglobulin, eGFR, PaO_2_/FiO_2_, and CD8+ T-cell counts.

The consideration of one to two of our observed risk factors (HR = 10.644; 95% CI: 2.305–49.159; *p* = 0.002) and all three risk factors (HR = 252.368; 95% CI: 24.390–2611.295; *p* < 0.001) were found to be independently associated with progression to severe COVID-19 (Table 6) during 14 days of hospitalization after admission.

**Table 6:** Independent risk factors for progression to severe COVID-19

	Total	Progression to severe COVID-19	*p*	HR	95% CI	*p*	Adjusted HR	95% CI
Number of factors (age > 65, CK ≥ 180, CD4+ cell counts <300)								
0	50	2	1				1	
1–2	31	11	0.02	10.967	2.428–49.532	0.002	10.644	2.305–49.159
3	2	2	<0.001	110.007	13.298–910.009	<0.001	252.368	24.390–2611.295
Unknown	33	2	0.677	1.517	0.214–10.771	0.490	2.006	0.277–14.497

CK: creatine kinase; adjustment variables: symptoms at admission, body mass index, ribavirin use, lopinavir/ritonavir use, antibiotics use, dyspnea, comorbid diabetes, comorbid hypertension, platelet count, C-reactive protein, lactate dehydrogenase, aspartate aminotransferase, albumin, beta-2 microglobulin, estimated glomerular filtration rate, partial arterial oxygen concentration/inspired oxygen faction, CD8+ cell counts.

### 3.7 Fourteen-day cumulative survival without developing severe COVID-19

We analyzed the period from admission to developing severe COVID-19 over the duration of 14 days by the Kaplan–Meier method. We found that in patients aged 65 years or older, the rate of not progressing to severe COVID-19 at the end of 14 days was 46.2%, whereas in patients younger than 65 years, the rate of not progressing to severe COVID-19 at the end of 14 days was 90.2%, and the calculated difference in the rates of severe COVID-19 development between the two groups of patients was found to be significant in the statistical analysis.

**Figure 1:**
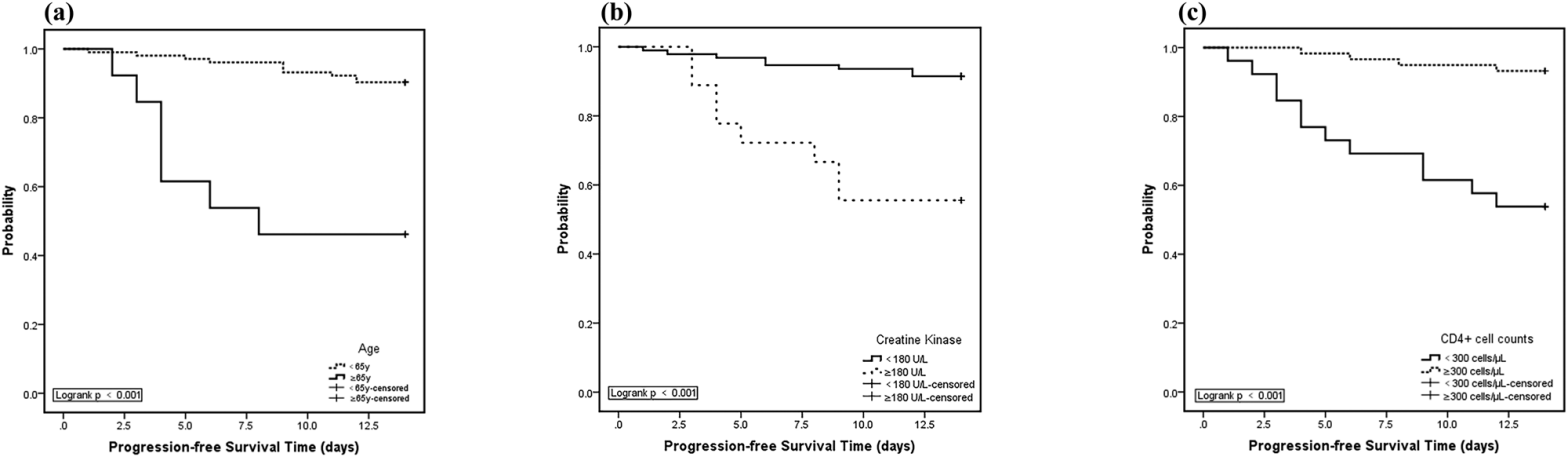
Kaplan–Meier curves for rates of not developing severe COVID-19 during 14 days after hospital admission. (a) Kaplan–Meier curves showed worse progression-free survival rates for COVID-19 patients aged 65 years or older compared to patients younger than 65 years (*p* < 0.001, two sided); (b) Kaplan–Meier curves showed worse progression-free survival rates for COVID-19 patients with CK ≥180 U/L compared to patients with CK <180 U/L (*p* < 0.001, two sided); (c) Kaplan–Meier curves showed worse progression-free survival rates for COVID-19 patients with CD4+ T-cell counts <300 cells/µL compared to patients with CD4+ T-cell counts ≥300 cells/µL (*p* < 0.001, two sided).

The following findings were also observed in our analysis. In patients with a CK ≥ 180 U/L, the rate of not progressing to severe COVID-19 at the end of 14 days was 55.6%, whereas in patients with a CK < 180 U/L, the rate of not progressing to severe COVID-19 at the end of 14 days was 91.5%, and again, there was a significant statistically calculated difference between these two rates of progression. In patients with CD4+ T-cell counts <300 cells/µL, the rate of not progressing to severe COVID-19 at the end of 14 days was 53.8%, whereas in patients with CD4+ T-cell counts ≥300 cells/µL, the rate of not progressing to severe COVID-19 at the end of 14 days was 83.2%, and the statistical difference between these two groups of patients was, again, computed to be significant (Figure 1).

## 4 Discussion

This study investigated the risk factors for progression to severe COVID-19 in patients diagnosed as mild or moderate COVID-19. The median age of patients developing severe COVID-19 during the hospitalized period of 14 days was significantly higher than that of those not developing severe COVID-19 (59 years [IQR, 50–70] vs 41 years [IQR, 35–54], *p*  < 0 .001), which concurs with the study results published previously [9].

In our study cohort of patients with milder COVID-19, we failed to observe a significant association between the presence of chronic diseases and the risk of disease progression, suggesting that the presence of chronic diseases may not necessarily contribute significantly to disease severity progression in such patients. Previous studies, however, have observed that some underlying chronic diseases, including hypertension and diabetes, may be risk factors for poor prognosis of COVID-19 [10,11,12]. The poor correlation of our results compared to that of other studies may be secondary to dissimilar study populations, differing sample sizes, and results obtained at different stages and locations of the COVID-19 outbreak, and warrants further investigation.

As outlined in Table 2, we did not observe any association between clinical symptoms and risk of disease progression. Frequently reported symptoms in our cohort of patients included fever, cough, sputum production, and fatigue. There were no significant differences in the proportion of patients progressing to severe COVID-19 between patients who exhibited the aforementioned symptoms and those who did not, suggesting that these symptoms were not sensitive indicators for disease progression in milder COVID-19 patients.

Compared with patients not developing severe COVID-19 during the period of 14 days after admission, patients progressing to severe COVID-19 during this period were more likely to have been administered antibiotics (10.1% vs 35.3%, *p*  = 0.016). Antibiotics were used in this subgroup of patients to prevent or treat secondary nosocomial bacterial infections. Our result indicates that antibiotic use may not be useful in arresting disease progression in the natural history of COVID-19.

In the present study, we found that age ≥65 years, CK ≥ 180 U/L, and CD4+ cell counts <300 cells/µL at admission were associated with disease progression during 14 days after hospital admission in patients with milder COVID-19. This result concurs with the previous study results in patients with severe acute respiratory syndrome (SARS), Middle East respiratory syndrome (MERS), and COVID-19, in which older age was also found to be a risk factor for progression to severe disease [13,14,15,16,17]. Similarly, a recent study from Wuhan also found that laboratory cardiac injury diagnostic parameters, including CK, were associated with poor prognosis in COVID-19 patients [18]. This has also been observed in patients developing severe SARS and MERS, who also tended to have significantly higher CK levels (≥180 U/L) [19,20]. Secondary systemic myositis as a direct consequence of coronavirus infection may be a reasonable explanation for this increase in CK levels [21]. In addition, a decline in CK levels has been significantly associated with COVID-19 mRNA clearance ratios, which may indicate that this may be a good indicator for recovery of COVID-19 infection [22]. Wong et al. reported that T-lymphocyte subsets may be depleted early in the course of SARS and that low levels of CD4+ T-cell and CD8+ T-cell counts may be associated with poor clinical outcomes [23]. In the present study, we also observed that a CD4+ T-cell count <300 cells/µL was an independent risk factor for progression to severe COVID-19, suggesting that patients with milder COVID-19 develop CD4+ T-cell count depletion before significant disease progression, which was similar to the study conducted in Shanghai [17].

Our study has limitations. First, as a retrospective, observational study, it is inevitable that some data were incomplete, and this could possibly have led to biased effect estimate results. Second, the study period for data observation was only 14 days, which may not have been a long enough period to reflect actual disease progression during the course of the natural history of COVID-19. Third, the number of different factors included in our study for univariate and multivariate analyses may not have been comprehensive enough, and some potential risk factors may have been missed. Despite these limitations, our results may nevertheless be useful to indicate potential markers for possible disease progression in patients with mild to moderate COVID-19.

We observed that age ≥65 years, CK ≥ 180 U/L, and CD4+ T-cell counts <300 cells/µL at admission were risk factors associated with disease progression to severe COVID-19 during 14 days after admission. These factors may represent potential markers for possible disease progression in patients with milder COVID-19. Affording due attention to these risk factors may facilitate early identification of patients with the potential for progression to severe COVID-19 in the mild and moderate COVID-19 patient population. Patients with these risk factors will require close monitoring for potential COVID-19 disease progression during their hospital admission.

## Abbreviations

ALT: Alanine aminotransferase
AST: Aspartate aminotransferase
BMI: Body mass index
CK: Creatine kinase
COVID-19: Coronavirus disease 2019
eGFR: Estimated glomerular filtration rate
HR: Hazard ratios
ICU: Intensive care unit
IQR: Interquartile range
LDH: Lactate dehydrogenase
LPV/r: Lopinavir/Ritonavir
MERS: Middle east respiratory syndrome
PaO_2_: Partial pressure of oxygen
PaO_2_/FiO_2_: Partial arterial oxygen concentration/inspired oxygen faction
SARS: Severe acute respiratory syndrome
SARS-CoV-2: Severe acute respiratory syndrome coronavirus 2
SD: Standard deviation
TCM: Traditional Chinese medicine
